# A Parallelized, Automated System for Higher-Throughput Spatial Navigation Testing of Rats

**DOI:** 10.1523/ENEURO.0415-25.2026

**Published:** 2026-08-21

**Authors:** Violet T. Saathoff, Josiah A. Quinn, Zhana D. Prince, Charles I. O’Leary, Anna K. Gillespie

**Affiliations:** ^1^Department of Neurobiology and Biophysics, University of Washington, Seattle, Washington; ^2^Graduate Program in Neuroscience, University of Washington, Seattle, Washington; ^3^Department of Lab Medicine and Pathology, University of Washington, Seattle, Washington

**Keywords:** aging, automated, electrophysiology, hippocampus, learning, memory, neurophysiology, parallelized, rats, rodents, spatial navigation

## Abstract

Spatial navigation tasks for rodents have been an extremely useful tool for studying cognitive processes for decades but are often laborious to administer and sensitive to experimenter-induced variability. To address these limitations, we have developed a parallelized maze system which runs four highly flexible maze environments simultaneously with automated reward delivery, enabling reduced experimenter involvement, increased throughput, and streamlined data collection. Further, our software tracks subject performance and manages the progression through a preset curriculum as subjects learn, reducing experimenter error and ensuring comparable learning curves across cohorts. Equipped with IR beam break–based reward ports, peristaltic pumps for liquid reward delivery, and overhead video tracking, our current maze accessories can be flexibly reconfigured to enable a vast array of behavioral tasks and support a wide range of experimental questions. Pilot cohorts of young (4–8 months) and aged (28–32 months) male and female rats demonstrate that this apparatus enables the efficient training of a series of increasingly difficult visually cued and memory-dependent spatial navigation task variants across a wide range of performance abilities.

## Significance Statement

To increase the efficiency and reliability of spatial navigation task administration, we have designed a behavioral apparatus which enables the training of four subjects in parallel on a variety of tasks with automated reward delivery. Further, we developed a software to track subject performance and advance training parameters through a preset curriculum to reduce experimenter error and enable robust comparison of learning metrics across cohorts.

## Introduction

For decades, scientists have studied the behavior of rodents navigating maze-like environments in order to understand diverse cognitive processes such as memory, decision-making, inference, fear, regret, sociability, and many more (Tolman, 1948; Olton and Samuelson, 1976; Morris, 1981; Steiner and Redish, 2014; Wijnen et al., 2024). Spatial navigation tasks for rodents have been particularly powerful tools due to their ethological relevance—a hungry rat will reliably seek food in a complex environment, just as it would in the wild. Spatial navigation tasks can provide a rich and sensitive behavioral readout of neural computations because they often incorporate multiple cognitive components: for instance, a subject might need to identify its location in an environment; recall past locations of food, obstacles, or threats; identify the most valuable location; plan a route toward that goal; execute the movement; and evaluate the outcome of that choice (Wijnen et al., 2024). Depending on the task, this might require using multiple types of memory—declarative, procedural, and/or working memory. Due to their sensitivity and reliability, spatial navigation tasks have become the gold standard for characterizing cognitive impairments in the case of neurodegenerative and other disease models (D’Hooge and De Deyn, 2001; Astur et al., 2002; Vorhees and Williams, 2014). Clever experimental design can allow researchers to tease apart the different cognitive components of spatial navigation challenges, and by incorporating simultaneous neural recording approaches, researchers have been able to begin describing the neural mechanisms that underlie these cognitive processes. Perhaps the most well-known finding stemming from this approach was the discovery of spatially tuned neurons in the hippocampus (O’Keefe and Dostrovsky, 1971), which has since inspired decades of research into the neural activity underlying the encoding of spatial experiences and their subsequent storage as memory (Moser et al., 2015; Findlay et al., 2020). However, there is still much that we do not understand about how the brain enables spatial navigation behavior. Thus, many further experiments, involving creative task variants, different neural recording modalities, and cohorts of different ages, sexes, and disease conditions remain a high priority.

However, spatial navigation tasks have some notable limitations which can slow progress. They are typically large and often designed for a single behavioral task, limiting the utility of each piece of equipment. Subjects are usually run serially, limiting behavioral cohort sizes and making behavioral testing slow and laborious. A human experimenter is often an essential part of the training/experimentation process (e.g., delivering a food reward or providing an escape opportunity in an aversive condition), adding an experimental variable which is hard to track and which limits the scalability of behavioral testing (Chesler et al., 2002; Pioli et al., 2014). When combined with neurophysiology, the need for additional subject space, tethering, or overhead tracking can place additional constraints on maze environments. Finally, because these experiments can be so costly and time-consuming, subjects are often selected for their motivation and aptitude for the task to maximize the chance of successful data collection. As a result, these cohorts may be biased toward high performers rather than reflecting the extent of natural variability in the population.

We set out to develop a spatial navigation maze apparatus that would address many of these limitations. Specifically, we set out to meet the following five criteria. First, we wanted to be able to run multiple subjects at once in order to increase efficiency, enabling larger cohort sizes (Poddar et al., 2013; Hao et al., 2021). Second, we wanted to automate the task in order to minimize experimenter-induced variability (Chesler et al., 2002; Pioli et al., 2014; Qiao et al., 2019; Zou and Li, 2019). Third, we wanted the apparatus to be flexible enough to run a variety of tasks (Porter et al., 2025). Not only would this provide more opportunities to use the same equipment for a range of experimental questions, but it would allow us to design a task curriculum that spans a range of difficulty, progressing from an “easy” task variant to more challenging ones so that we could run structured, systematic behavioral testing across the full range of individual abilities (Holleman et al., 2019). Fourth, our apparatus needed to be compatible with wireless neurophysiological recordings. For hippocampal recordings to be analyzed in relation to spatial location, this required overhead position tracking as well as sufficiently wide corridors for an implanted rat. The training curriculum (including initial task acquisition) would ideally take <4 weeks, a time window similar to our periods of high-quality neural recording signal. Finally, our behavioral task also needed to be highly structured, to enable the collection of many comparable behavioral trials and ensure good spatial coverage of the entire environment for subsequent analysis.

Our resultant maze apparatus is a low-cost, scalable, highly automated, and parallelized setup that can be configured to run a variety of different spatial navigation tasks. Our proof-of-principle apparatus allows experimenters to administer spaced behavioral training sessions to four rodent subjects simultaneously. The behavioral arenas described here are designed to be consistent, efficient, and simple to construct. The logic of the task can be easily customized; here we focus on a task which includes both visually cued and memory-dependent variants. We have developed a Python package that automates the advancement through a series of task variants for each subject based on set performance criteria. This highly structured, automated training program reduces the opportunities for experimenter error and ensures that all subjects attain certain criteria before advancing, providing comparable behavioral epochs within which to study neural mechanisms of learning and performance. As much as possible, our system relies on open-source software packages and hardware designs. All custom designs, components, and software are shared here as a resource (Table 1), in addition to extensive instructions and part lists, to make this system easy to scale, replicate, and implement.

**Table 1.** Resources for creating and running the maze apparatus

Maze code	Main scripts
	Modules
Maze design	Maze CAD
	Laser-cutter drawings
	Reward port CAD
	Reward port PCB
	Pump plate CAD
	Pump controller PCB
	Bill of materials

## Materials and Methods

### Design overview

**Figure 1.**
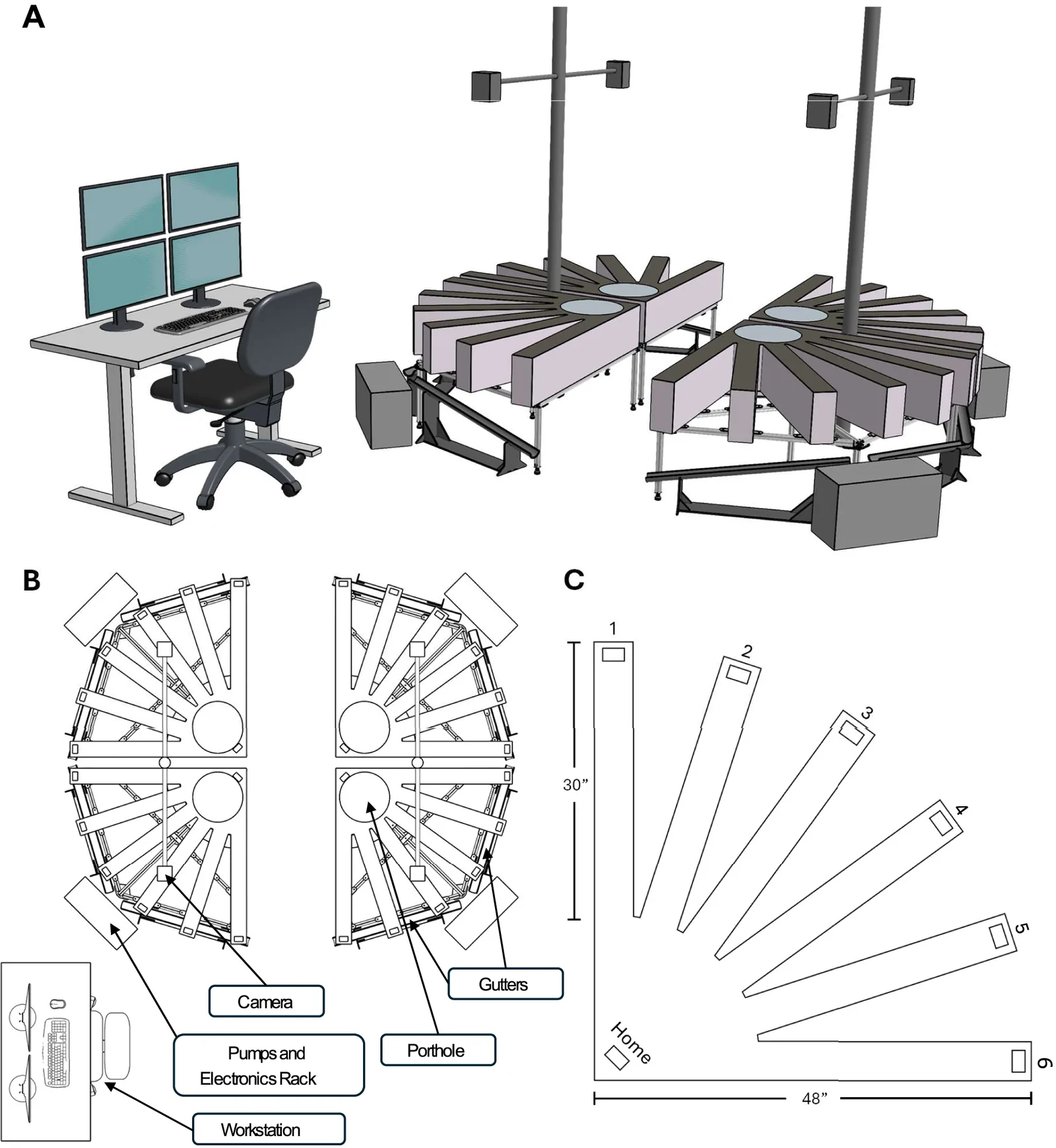
Room overview. ***A***, 3D view of the entire room. Note that colors/opacity of various components are not true to the actual design, and are instead intended to help with visual clarity. ***B***, Top-down view of the entire room, showing the layout of all four maze environments, as well as the workstation (bottom left). ***C***, Top-down schematic view of a single maze environment, with reward ports indicated by rectangles. Additional photographs of the room setup and maze apparatus are included in Extended Data Figure 1-1.

The maze apparatus consists of four fully enclosed 6-arm maze environments overlooked by a centralized control station (Fig. 1*A*,*B*; Extended Data Fig. 1-1*A*). Each maze environment consists of a central home area with a single reward port and six radial arms, each with a reward port at the end (Fig. 1*C*). Each maze is equipped with an overhead camera, a pumps and electronics rack (PER), and a drainage system to handle liquid waste. At the control station, the experimenter can view the overhead video from each maze, control the behavior of the task, and monitor performance in real time (Extended Data Fig. 1-1*B*). From inside the maze, subjects can view both local intramaze cues and distal cues on the ceiling, room walls, and floor due to the transparent acrylic floor, ceiling, and mesh arm lids (Extended Data Fig. 1-1*C*). Whether the distal cues are used to guide behavior is outside the scope of this study but could easily be tested in the future by limiting the external view through the walls and ceiling with contact paper or other means of visual obstruction.

10.1523/ENEURO.0415-25.2026.f1-1 Figure 1-1 **Room Overview** A. Photograph of the entire room. B. Experimenter view of the 4 monitors, one per maze. On each monitor, the experimenter can view the overhead camera video, a live plot of be- havioral performance, streaming behavioral variables in the SpikeGad- gets Trodes software, and the SpikeGadgets StateScript behavioral con- trol module. C. Rat's-eye view from inside the maze closest to the command station, looking outward from the home port towards the desk/closest wall. Note the clear view through the maze lids of distal cues (the ceiling and walls) as well as features within the maze (8020 below the clear floor, corners, and ports at the end of each arm). Download Figure 1-1, TIF file.

### Maze construction

**Figure 2.**
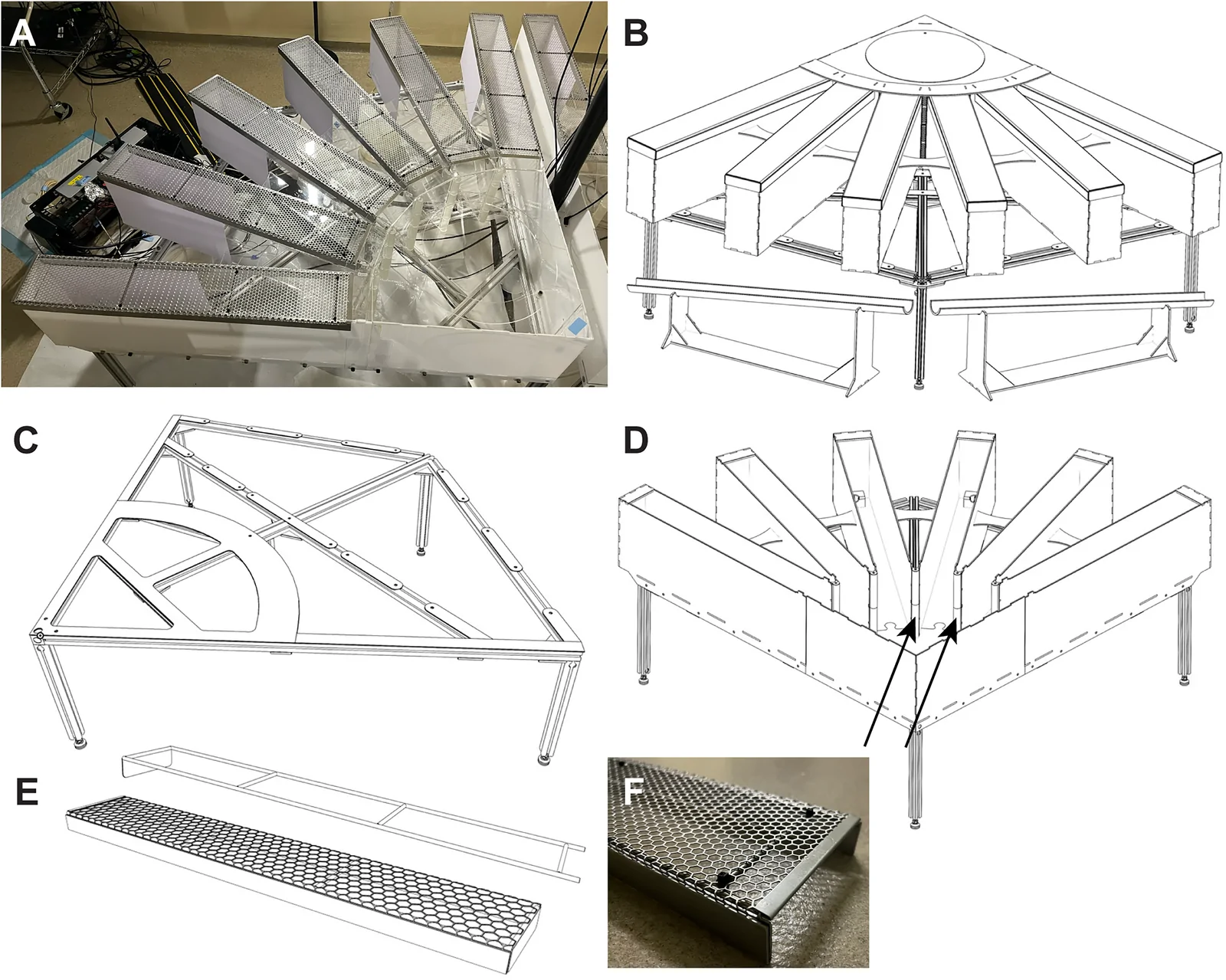
6-arm maze. ***A***, Photograph of the fully assembled maze (side) with lids and ceiling included. ***B***, 3D view of the fully assembled maze (back), with the drainage gutters clearly visible. ***C***, 3D view of the base (side). The base is made from 8020 extruded aluminum, and has laser-cut, 1/16″ acrylic tabs bolted to it. These tabs are then glued to the floor of the maze to help secure it to the 8020 base. ***D***, 3D view of the maze (front) with the lids and ceiling removed. Note the 3D-printed corner pieces (arrows) which join the walls between adjacent arms. ***E***, 3D view of the frame for an arm lid (top) and the complete arm lid (bottom). The frame adds rigidity and robustness to the hexagonal metal mesh lids, which helps to prevent them from becoming damaged over time due to normal use. Besides this panel, lids are displayed as being solid when in reality they are made of a hexagonally perforated aluminum affixed to a stainless steel frame. ***F***, Photograph of the maze lids to show the texture of the hexagonal mesh.

The environment is primarily constructed out of laser-cut acrylic (Fig. 2*A*,*B*) which rests on an 8020 extruded aluminum base (Fig. 2*C*). Acrylic was selected because it comes in a variety of colors (both transparent and opaque) and because we can manufacture and assemble the parts in-house easily and consistently. Where pieces are joined at 90° angles, box joints were used to carefully and reliably control the positions of various components. The floor of the maze is made out of 3/16″ clear acrylic which provides satisfactory rigidity. The transparent floor did not appear to cause any anxiety in rat subjects; they freely explore immediately when placed in the environment and did not show any preference for maze areas with an opaque floor. Maze walls were made from 1/8″ acrylic, which provides sufficient rigidity. Most of the walls were made from clear acrylic to create a complex visual environment, although adding matte contact paper around the ends of each arm was helpful for minimizing potentially confusing reflections of illuminated reward ports. The outer walls were made from white acrylic to prevent the subjects from seeing each other.

The environment is fully enclosed to prevent subjects from escaping, with a ceiling height of 9″. The ceiling over the home area is made of clear 3/16″ acrylic, with a 10.5″ porthole for putting subjects into the maze and a clear 1/8″ acrylic porthole cover (Fig. 2*A*,*B*). The ceiling over each arm is a removable hexagonally perforated aluminum lid, affixed onto a stainless steel rod frame (Fig. 2*A*,*E*,*F*). The perforated lids allow for air to enter the environment, introduce only minimal occlusion for overhead camera tracking, and are easily removable to allow the experimenter to access maze arms when needed; all exposed perforations were hemmed to protect both the experimenter and the subject from sharp edges. To secure the lids, one lid end slides under an overhang in the ceiling of the home area, while the outer end has an end cap added. While a fully enclosed design does prevent tethered recordings, minimizing experimenter supervision was critical for successfully running parallelized behavior.

For the maze base, we selected 8020 extruded aluminum framing as it is modular, strong, and easily attainable, easily supporting the maze while giving us options for routing tubing, cables, and drainage (Fig. 2*C*). To prevent undue stresses in the acrylic floor which could cause eventual cracking, care was taken to ensure that the outer rails of the frame were positioned as close to 90° as possible and that the feet of the 8020 base distributed the weight of the maze as evenly as possible. To secure the maze to the base, the main body of the maze was epoxied to a layer of 1/16″ acrylic, which was then bolted to the 8020 base (Fig. 2*C*). Additionally, the outer walls of the maze were themselves bolted directly to the 8020 base, providing a very solid connection between the base and the maze. Acrylic pieces were bonded using a clear, slow-cure 2-part epoxy (BSI-205), or occasionally with acrylic cement (Weld-On 3). For better gluing surfaces, the walls of adjacent arms are joined with 3D-printed corner pieces (Fig. 2*D*, Extended Data Fig. 1-1*C*).

### Reward ports

**Figure 3.**
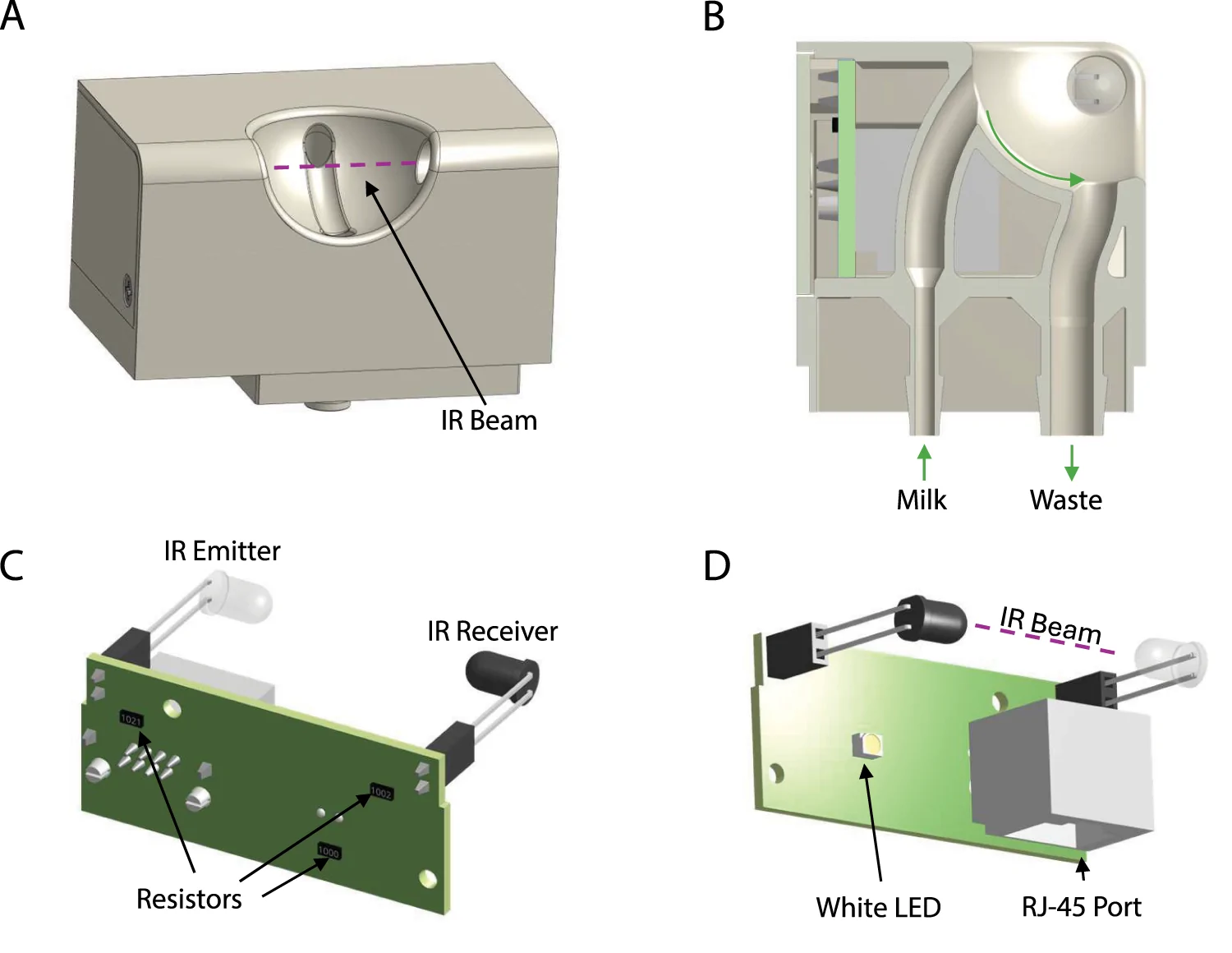
Reward delivery. ***A***, Reward port adapted from Roumis (2020), front view. When the rat's nose enters the cavity, the IR beam is broken, and the port is “triggered.” This behavioral event can trigger milk delivery and/or other environmental actions. ***B***, Cutaway view, showing the flow of liquid through the reward port. Milk enters the port through a 1/8″ tube and drains out through a 1/4″ tube, both connected to the well with a barb fitting. ***C***, Reward port PCB, back view. ***D***, Reward port PCB, 3D front view. The RJ-45 port allows the port to be connected to the pump control electronics via an Ethernet cable, enabling readout of the IR beam state and control over the state of the white LED.

The most critical features of the maze are the reward ports, which dispense a liquid food reward (evaporated milk with 5% sucrose added). Each port consists of an IR photogate (emitter and receiver; 890 nm), a white flat-mount LED, a 1/8″ inner diameter inlet port, a 1/4″ inner diameter outlet port, and an RJ-45 port for communication with the environmental control unit (ECU; Fig. 3*A–D*). The reward ports' body and their electronics are based on an established design (Roumis, 2020). Compared with the original design, barbed tube fittings were added to make it easier to connect tubing to the reward ports, the drainage port was enlarged to help avoid overflows during cleaning, and the footprint was streamlined to remove crevices where refuse could easily accumulate. The body of the reward ports was printed on a Formlabs Form3 printer using clear resin, and a custom PCB is populated and installed inside. Printing with clear resin allows the white LED to shine through the translucent body of the reward port, allowing the port to illuminate and serve as an easily visible cue. When a subject breaks the IR beam, a signal is recorded and can trigger the delivery of liquid through the port via an associated peristaltic pump.

### Pumps and electronics rack

**Figure 4.**
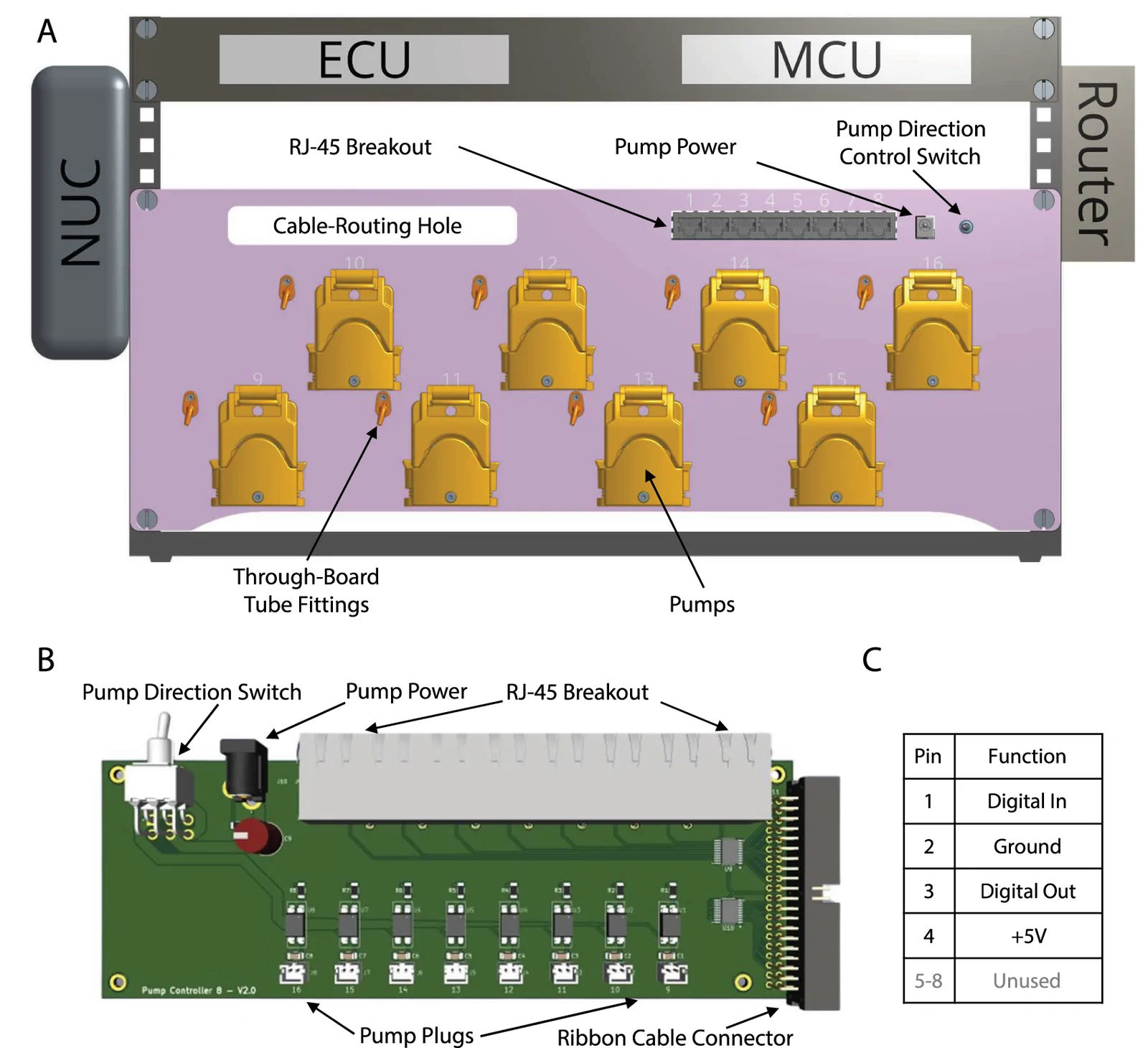
Pumps and Electronics Rack (PER). ***A***, Front view of the PER; a server rack which integrates the computer (NUC), the SpikeGadgets Main Control Unit (MCU), the SpikeGadgets Environmental Control Unit (ECU), and the pump plate (pink). The pump plate, made from 4 mm anodized aluminum, can hold up to eight Boxer 9QX-9007.730 peristaltic pumps and mounts a pump controller PCB. The pump control electronics also include an RJ-45 breakout board which routes reward port and camera sync signals. Tubing (data not shown) routes liquid from a single 1 L reservoir to each of the pumps and then through the pump plate via the through-board tube fittings to the reward ports. ***B***, Top-down view of the pump controller/RJ-45 breakout PCB. Each board connects to the ECU via a ribbon cable, and can individually power up to 8 pumps via the JST 2-PH connectors. Each board can also connect to eight accessories (typically reward ports) via Ethernet cables. ***C***, Pinout for each RJ-45 breakout port. When a breakout port is connected to a reward port, Pin 1 represents the state of the IR beam, Pin 3 controls the output of the white LED, and Pin 4 powers both the IR LED and the IR receiver. Example electrophysiological recordings demonstrating the lack of electrical noise due to the pumps in Extended Data Figure 4-1.

Each maze includes several key electronic accessories, which we combine into a single organized 4U server rack and refer to as the PER (Fig. 4*A*). The PER houses a compact computer (Intel NUC), a router for connecting to the overhead cameras, a pump control/reward port breakout board, and peristaltic pumps for liquid reward delivery. It also houses a neural data acquisition system. We chose products from SpikeGadgets (an Environmental Control Unit (ECU) and either a Main Control Unit (MCU) or LoggerDock) for their price point and data collection capabilities, but other systems such as Open Ephys (Siegle et al., 2017), Arduino, BControl (Bcontrol, 2025), or Bonsai (Lopes and Monteiro, 2021) environmental control strategies could be used. Each PER is connected to the central command station via the NUC, which sends display and peripheral signals to a KVM switch so that a single keyboard/mouse can control any of the maze stations. The PER for each maze is located close to the maze to minimize tubing/cable lengths and to minimize the tripping hazard that tubing and cables can cause. A custom mounting plate for the pumps and the pump control PCB was designed to mount to the server rack and secure all pumps, mitigating any vibration caused by pump activity.

10.1523/ENEURO.0415-25.2026.f4-1 Figure 4-1 **Electrophysiology During Maze behavior** A Top: single example of hippocampal LFP traces (tetrodes targeted to dorsal CA1 cell layer) during maze behavior, aligned to the onset of reward delivery at the home port. Bottom: rat speed over this time period, showing deceleration prior to and stopping at the reward port. B Top, hippocampal LFP traces averaged over all home port visits within a behavioral epoch, aligned to the onset of reward delivery. Bottom, raster plot illustrating the time of beam break (blue) and reward delivery (red) across all home port visits. C Top: single example of hippocampal LFP traces (tetrodes targeted to dorsal CA1 cell layer) during maze behavior, aligned to the onset of reward delivery at the outer arm ports. Bottom: rat speed over this time period, showing the deceleration and stopping at the reward port. D Top, hippocampal LFP traces averaged over all outer arm port visits within a behavioral epoch, aligned to the onset of reward delivery. Bottom, raster plot illustrating the time of beam break (blue) and reward delivery (red) across all outer arm port visits. Data visualization enabled by Figpack (Magland 2025). Download Figure 4-1, TIF file.

We use peristaltic pumps to deliver small amounts of liquid reward to reward ports. After extensive testing of many pump models, we selected Boxer 9QX peristaltic pumps. When delivering rewards, the ECU sends a TTL pulse train signal to the pump controller, which then modulates the output of solid-state relays which gate power to each pump. Pumps are powered using a standard 12V/2A DC power supply, which is routed through a switch to control the pumps' direction. While the pump controller is compatible with any pump that uses a standard DC motor (i.e., not a stepper motor or a DC brushless motor), the aluminum pump plate that integrates the pump controller and the pumps is specifically designed for the Boxer 9QX pumps. To control the pumps, we designed a pump control PCB (Fig. 4*B*) which interfaces directly with the SpikeGadgets ECU and operates as a breakout board for controlling maze accessories (all digital outputs are buffered). DIO ports 1–8 are each connected to an RJ-45 port (pinout in Fig. 4*C*), which we typically connect to the reward ports, but are fairly generic and can be used in a variety of ways. Ports 9–16, meanwhile, control the solid-state relays used to gate power to the pumps, with filtering capacitors to protect the board from back EMF. By using standard JST-PH 2 connectors to connect to the peristaltic pumps, this board can be easily used to modulate power to a range of devices as necessary. Importantly, we have not observed electrical artifacts caused by the pumps on neural recordings made in close proximity to the PER (Extended Data Fig. 4-1*A–D*). The pumps do make an audible noise when operating (Movie 1). However, especially after maze acclimatization, subjects do not show any signs of attending to pump sounds from either their PER or neighboring PERs, such as interruptions in movement trajectories, extended pauses, or changes in orientation. To minimize any potential cross-maze sound impacts, or if the task itself required sound cues, isolation might be achieved by installing soundproof barriers between each maze.

**Movie 1.** Room overview. Video taken from near the command station showing four male subjects participating in behavioral training (Step 1 task variant) simultaneously. The pump sounds from each maze apparatus are audible. [View online]

Critically, in order to achieve consistent reward delivery of an appropriate amount of milk at an appropriate speed, we designed a variety of pulse trains rather than simply turning the pump on for a precise duration. This modulates the duty cycle and results in a slower, more consistent milk delivery. For ∼30 µl “small” reward, we used three 10 ms pulses separated by 20 ms (33% duty cycle). For ∼130 µl “large” reward, we used ten 10 ms pulses separated by 20 ms. In addition to increasing consistency, this strategy is preferable to slowing the pumps by modulating their voltage, because it retains the ability to run pumps at full speed during milk loading and cleaning. All the pumps for each maze draw from a single 1 L reservoir of milk, streamlining the milk loading process and reducing waste. In addition, it greatly increases the efficiency of the cleaning process. After daily behavioral training is complete, pump direction is reversed via a switch on the pump control PCB, which allows us to collect the milk for overnight storage at 4°C. We then fill the reservoir with cleaning solution, return the pumps to their default direction, flush the tubing until clean, run ethanol through the tubing to sanitize, and then let it dry.

### Drainage

In an attempt to reduce the number of refuse containers, gutters were made so that up to three reward ports could share a single waste container (Figs. 1*A*, 2*B*). Additionally, some containers could be shared between two gutters, or between two home ports, reducing the total number of refuse containers for all four mazes to eight. Gutters were made from 2″ ABS tubing, cut lengthwise, and epoxied to a simple acrylic frame. It is critical to ensure that the tubing leading from the reward ports to the gutters maintains a sufficiently steep angle so that liquid does not pool and clog. Alternatively, longer tubes leading to shared refuse containers may also be used in place of gutters as long as the tubing angle is sufficiently steep.

### Maze assembly and affordability

A guiding principle of our maze apparatus is affordability. By designing a maze environment that could be consistently assembled in-house from laser-cut acrylic pieces, we avoided the high costs of custom manufacturing by commercial vendors such as Tap Plastics, Maze Engineers, Med Associates, or similar. In sum, the cost of materials for a single maze environment (acrylic, 8020, and hardware) came to ∼$1,000 and could be assembled in <1 d. In comparison, a comparable custom acrylic environment from a commercial vendor was quoted over $10,000. We use 3D-printed reward wells instead of purchasing nosepokes from Med Associates or similar. These can be ordered from online 3D printing companies such as Protolabs or Shapeways for ∼$90 each or printed with entry-level 3D printers in labs or makerspaces for even lower cost (in our case, $10 in resin each on a lab Form3 printer). The PER includes a neural data acquisition system (SpikeGadgets), which is a substantial investment for any lab doing neurophysiology, but could be optional for running behavior if another behavioral control system is used. The NUC computers are highly affordable ($400 each) compared with typical desktop computers. Peristaltic pumps, at ∼$140 each, are far more cost-effective than programmable syringe pumps (New Era Systems, $700 each) in addition to dramatically reducing cleaning time and effort. The overhead cameras are an exception to our budget design choices; the Allied Vision Manta cameras and lenses (together, $1,500 each) provide exceptional time synchronization abilities but could certainly be replaced with more affordable options if desired. Altogether, we estimate an up-front cost of ∼$15,000 for the complete 4-maze apparatus, command station, and all accessories.

### Maze task and curriculum

The spatial memory task that we ultimately wanted subjects to perform is a challenging memory-dependent “win-stay/lose-switch task,” related to prior work (Gillespie et al., 2021). Reliable performance is best achieved by using several simpler task variants (“steps”) to gradually shape behavior toward the final task (Holleman et al., 2019), which we refer to as our “curriculum.” Each step of the curriculum is designed to build upon knowledge from previous steps, with advancement criteria designed to minimize both frustration and complacency in order to encourage engagement and effective learning. Well-defined advancement criteria enable automated advancement through the curriculum while also helping to standardize the learning process across subjects and cohorts. Breaking down the learning of the full task into simpler components is not only easier for the subject but also opens up the possibility of studying the neural correlates of the learning process with more structure and specificity (Shujah et al., 2024)—thus both the training steps and the final task are scientifically valuable. Furthermore, by ensuring that all subjects meet standardized performance thresholds before advancing to the next step of the training process, this approach allows us to quantify measures of performance at each step, giving us valuable insight into the learning process. We focus on our 6-arm task and its training steps here, but many other types of tasks could be implemented, each with their own training curriculum, within this or other maze environments.

### Task

**Figure 5.**
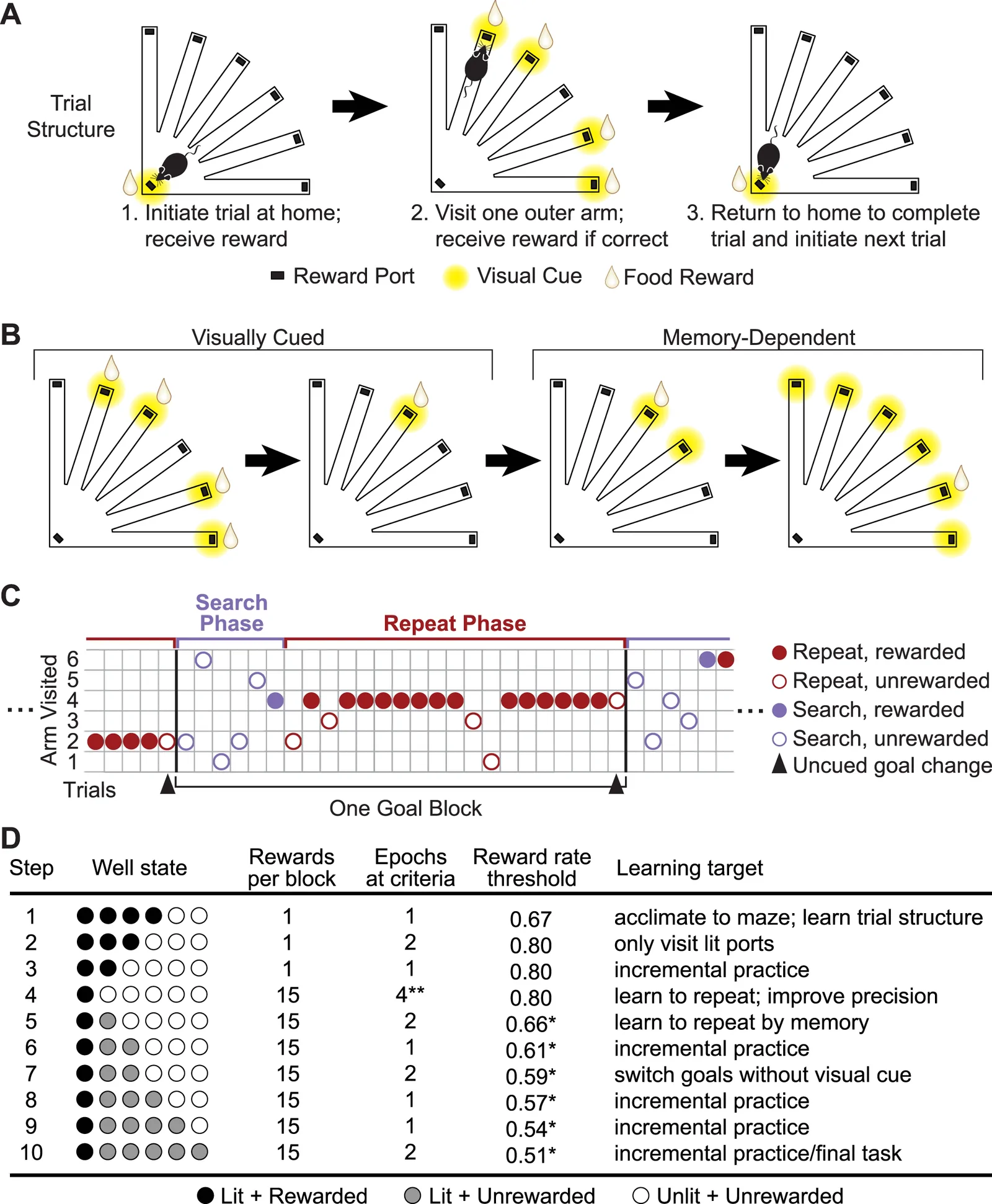
Training curriculum. ***A***, Visual schematic of a single example trial at Step 1, including the initiating poke at the home port, choice of one outer arm, and the return to home to complete the trial. ***B***, Visual schematic of training progression, illustrating Steps 1, 4, 5, and 10 of the training curriculum. ***C***, Example trial series for Step 10 behavior, illustrating the classification of search and repeat trials. ***D***, A table giving an overview of the 10-step training curriculum. Outer reward ports exist in one of three states: Lit + Rewarded, Lit + Unrewarded, and Unlit + Unrewarded. In Steps 5 and 6, when switching goal blocks, the previous goal well is never lit, giving the subjects a visual cue when it is time to switch goals. In Steps 7 through 10, however, the previous goal well is always lit (unrewarded), forcing the subjects to learn to switch goals on their own. * indicates goal rate thresholds that were used for the female cohort and are recommended. The thresholds for these steps for the male cohort were marginally higher: 0.70, 0.66, 0.63, 0.60, 0.58, and 0.55, respectively. ** indicates the recommended number of epochs at Step 4; only two were required for the male cohort.

For all 6-arm task steps, the subject must perform structured trials which consist of a nose poke at the home port, then a visit to a single arm of the maze, and then a return to the home port (Fig. 5*A*). Each trial begins when the subject triggers the illuminated home port and receives a small reward. Next, some number of the outer ports illuminate, and the subject must choose one to visit, receiving a large reward if the arm is a goal arm, and nothing otherwise. After visiting a single-arm port, the subject must return home to begin the next trial. The first few task steps are visually cued: rewarded outer arm ports are lit while unrewarded outer arm ports are not, so visiting any lit arm port will result in the subject receiving a reward (Fig. 5*B*,*D*). Later, subjects transition to memory-dependent task steps, during which both rewarded and unrewarded ports are lit, and subjects must discover and remember which one(s) deliver reward (Fig. 5*B*,*D*). In later task steps, goal arm locations are changed in a “block” structure (after 15 goal arm visits; Fig. 5*C*). Within each goal block, the “search phase” is the set of trials until the goal arm has been identified, and the “repeat phase” includes all subsequent trials; the search phase includes the first rewarded trial. When the goal block is complete, new goal arm(s) are drawn, and the subject must begin a search phase to find the new goal arm(s). Any deviation from the described trial structure, such as going from one arm port to another, initiates a “lockout,” where all wells are deilluminated/deactivated for a predetermined interval (typically 15 s). After the lockout is over, the home well illuminates, and the subject must return home to start the next trial. Depending on the maze parameters, the epoch ends after a set number of trials, a set number of goal blocks, or a set amount of time (typically a maximum of 70 min from the epoch start). At this time, all wells are automatically deilluminated/deactivated, and subjects are left to wait for the experimenter to remove them from the maze.

### Curriculum

Before beginning the 6-arm curriculum, subjects are first trained to trigger reward wells in order to receive milk reward on a simple linear track. Subjects must first earn *>*50 rewards in a 45–60 min training session before advancing to the maze; this typically takes 3–5 training sessions.

The full 6-arm curriculum consists of 10 increasingly difficult steps as outlined in Figure 5*D*. Each step in the curriculum is characterized by a set of parameters: the number of goal ports, the number of lit ports, the number of rewards per goal block, the number of trials in the epoch, and the goal rate threshold (#rewarded trials/#trials over the entire epoch). The number of goal ports determines how many ports will be chosen as goals each block. The number of lit ports is the total number of reward ports with their LEDs turned on during the outer arm phase of each trial. These ports are selected randomly once per block alongside the goal port and exist as all options the subject must explore when searching for the goal. Ports which are lit but unrewarded will never give a reward during the current goal block. The number of rewards per goal block determines how often the goal changes. Lastly, for an epoch to be considered “successful,” the subject must complete at least the minimum number of trials (typically 50 trials) while achieving a goal rate greater than or equal to the threshold goal rate. Goal rate thresholds for memory-dependent steps are generally calculated as 75% of the rate a perfectly trained subject would achieve if they were maximally unlucky, minus an additional 5% buffer for the memory-based steps. At each step in the curriculum, subjects must complete a specified number of successful epochs before moving on to the next step. After the completion of Step 10, subjects are considered fully trained, although additional epochs of behavior may be collected to fully characterize final performance levels.

### Curriculum implementation

Setting task parameters manually, especially with increasing cohort size and parallel training sessions, is extremely prone to experimenter error, while also being tedious, stressful, and time-consuming for the experimenter. To counter this, each subject's environment parameters and learning progress are stored in a text file in order to fully automate the subject's progress through the curriculum. This text file is associated with the subject's name and is automatically read/updated by the same Python script which controls the maze environment. In addition to being less error-prone, this strategy enforces uniform and objective environment parameter selection between training stages across subjects, removing any experimenter variability in deciding when to advance subjects to the next task step.

To safeguard against any errors which may occur during the epoch, the parameter file is updated as trials are completed, ensuring that it is as up-to-date as possible at all times. Furthermore, a copy of the parameter file is also added to the behavior log at the beginning and end of each epoch, which makes the parameter set accessible for downstream data processing. This also preserves a record of which changes were made to the file by the maze code and which changes were made by the experimenter between epochs (atypical, but occasionally necessary).

### Arm selection

Due to thigmotaxis, subjects tend to begin behavioral training with an innate bias toward arms 1 and 6, since those arms share a continuous wall with the home area. As subjects explore the maze and learn the task, they tend to develop idiosyncratic biases toward and against specific arms, which confounds behavioral analyses. To counter these biases, an arm selection algorithm was implemented that tracks the subject's arm visits and draws from a probability distribution which favors less frequently visited arms, encouraging subjects to visit all arms as evenly as possible.

The premise of the algorithm is to maintain a value for each arm which quantifies how much the subject has visited that arm as a proxy for the subject's arm biases and to then use this data to generate a probability distribution for arm selection which is counter to those biases (i.e., the more an arm was recently visited, the less likely it is to be selected as a goal arm, and vice versa). Goals are then selected from this distribution as needed.

There are four parameters which control the algorithm:

1. *α*: weights between rewarded and unrewarded visits. α∈[0,1]; α∼0.5 is recommended.
2. *β*: a “temperature” parameter which controls how aggressively the algorithm will push subjects to explore all arms evenly. β<0; β>−2 is recommended.
3. *γ*: a decay parameter which increases the responsiveness of the algorithm, causing more recent data to be weighted more heavily than older data (the data's relevance to arm selection decays exponentially). This decay is applied approximately once per epoch via a derived quantity, $\gamma^{\prime }=\gamma^{\;1/\mathrm{expected}\;\mathrm{trials}}$, which is applied after each trial. γ∈(0,1); γ>0.5 is recommended.
4. *δ*: a floor for the selection probability of each arm. If the algorithm ever pushes the probability of selecting a particular arm to effectively 0, subjects can and will overcorrect, learning to completely avoid that arm, which is hard to recover from (in general, we found it easier to train subjects away from their favorite arms rather than toward arms they were averse to). This floor parameter helps prevent overcorrection when subjects are pushed away from arms they previously had a strong bias toward. This is achieved by computing the derived quantity, $\delta^{\prime }=\delta /(1-6\delta )$, which is calculated such that $\mathrm{Min}(\left(\vec{P}+\delta^{\prime })/(1+6\delta^{\prime }\right))\geq \delta$, where $\vec{P}$ is a probability distribution array with 6 elements, and all calculations are done element-wise. δ∈[0,1/6); 0.01≤δ≤0.03 is recommended.

To implement the arm selection algorithm, subjects’ arm visits are tracked in an array called $\vec{\mathrm{weighted}\;\mathrm{visits}}$. This array is updated after each trial according to the following algorithm, where well is the index of the arm the subject visited this trial, and reward is 1 if the subject received a reward this trial, or 0 otherwise. All array computations are element-wise:

$$\vec{\mathrm{weighted}\;\mathrm{visits}}\to \;\vec{\mathrm{weighted}\;\mathrm{visits}}\times \gamma^{\;\prime }$$

$$\vec{\mathrm{weighted}\;\mathrm{visits}}\to \;\vec{\mathrm{weighted}\;\mathrm{visits}}[\mathrm{well}]+(\alpha \times \mathrm{reward}+(1-\alpha )(1-\mathrm{reward}))\times \gamma .$$

At the beginning of each goal block, arm(s) are selected at random based on a probability distribution generated from the $\vec{\mathrm{weighted}\;\mathrm{visits}}$ array using the following formula, where all array computations are element-wise:

$$\mathrm{softmax}(\vec{X},\beta )=\frac{e^{\beta \vec{X}}}{\sum e^{\beta \vec{X}}}$$

$$\vec{Q}=\mathrm{softmax}(\vec{\mathrm{weighted}\;\mathrm{visits}},\;\beta )$$

$$\vec{P}=\frac{\vec{Q}+6\delta^{\prime }}{1+6\delta^{\prime }}$$

When only one goal is being selected (Steps 4+ of the curriculum), no arm is ever selected as the goal arm twice in a row. After Step 6, when subjects are searching for a single goal arm among several lit options, we found that a simpler arm selection algorithm was more effective. Six goals are selected at a time, without replacement, and the goal schedule is stored in the parameter file to allow it to persist between epochs. When redrawing goals, no goal is ever selected twice in a row.

### Data outputs

For post hoc analysis, we need reliable records of all behavioral events, such as port triggers, port illumination state, and reward delivery times. Further, we need a system that is resilient to common experimenter errors such as typos in filenames and recording mistakes. We developed a system of multiple logs, each with varying levels of detail, which are initialized at the start of each epoch and which automatically close themselves at the end of the epoch.

The first output is a highly detailed log containing the maze parameters, initialization statements, and all maze events. Furthermore, it is preemptively segmented into goal blocks to make data processing as easy as possible. The second log is much more compact, containing one line for the maze parameters and a table of important trial events. Each trial contains the data for the home visit, the outer arm visit, goal block details, and the details for any lockout port visits. The data recorded for each port visit includes the time and duration of the IR beam break, which port was triggered, and whether or not a reward was delivered. While not as detailed as the full log, this format is extremely easy to process and requires no detective work to assemble the trial landmarks, making it very convenient for data processing.

Additionally, each subject's arm visits are plotted live at the control station, meaning that the experimenter can monitor all subjects’ progress simultaneously (Extended Data Fig. 1-1*B*). This both allows the experimenter to gain a deeper understanding of how subjects are performing the task without having to constantly watch the subjects and makes it easy to quickly detect any maze malfunctions. The videos collected by overhead cameras during behavior are also saved for post hoc position tracking or pose estimation as needed (Movies 2 and 3).

**Movie 2.** Overhead video, Step 4. Video taken from the overhead camera of one maze apparatus, showing a male subject being placed in the maze and performing a Step 4 (visually cued) training session. Video is 4× real speed; no audio. [View online]

**Movie 3.** Overhead video, Step 10. Video taken from the overhead camera of one maze apparatus, showing a male subject being placed in the maze and performing a Step 10 (memory-dependent) training session. Video is 4× real speed; no audio. [View online]

### Resources and code accessibility

As many aspects of this maze apparatus have been made open source as possible; links to solid models, laser-cutting drawings, and PCB design files can all be found in Table 1, along with a complete bill of materials. Additionally, the Python codebase used for task logic, performance tracking, and curriculum advancement is freely accessible (Saathoff and Gillespie, 2025a,b) without restrictions.

### Experimental model and subject details

To test the automated, parallelized maze apparatus, we trained cohorts of 8 male and 8 female Fischer × Brown Norway F1 rats from the NIA colony (Charles River). Each cohort included four young (4–9 months) and four aged (28–32 months) subjects. All animals were kept on a 12 h light/dark cycle (lights on 7 A.M.−7 P.M.) with *ad libitum* access to food (standard rat chow) in a temperature- and humidity-controlled facility. Beginning 1 week prior to training, rats were singly housed and food restricted to 85% of their free-feeding weight. Data were collected from over 32 weekdays (male cohort) and 37 weekdays (female cohort). Food restriction was maintained on weekends, but behavioral training was not administered. Each subject completed 2–3 epochs per training day. Subjects in each cohort were divided into two subgroups of four each, each containing two young rats and two aged rats; the subgroup which ran first in the day was alternated each day. Each subject was assigned to a specific maze, with one young rat and one aged rat assigned to each maze. Behavioral training for the male and female cohort was conducted serially, not interleaved, with thorough cleaning of the room between cohorts to avoid any overlap in odor cues. All procedures were approved by the Institutional Animal Care and Use Committee at the University of Washington.

## Results

**Figure 6.**
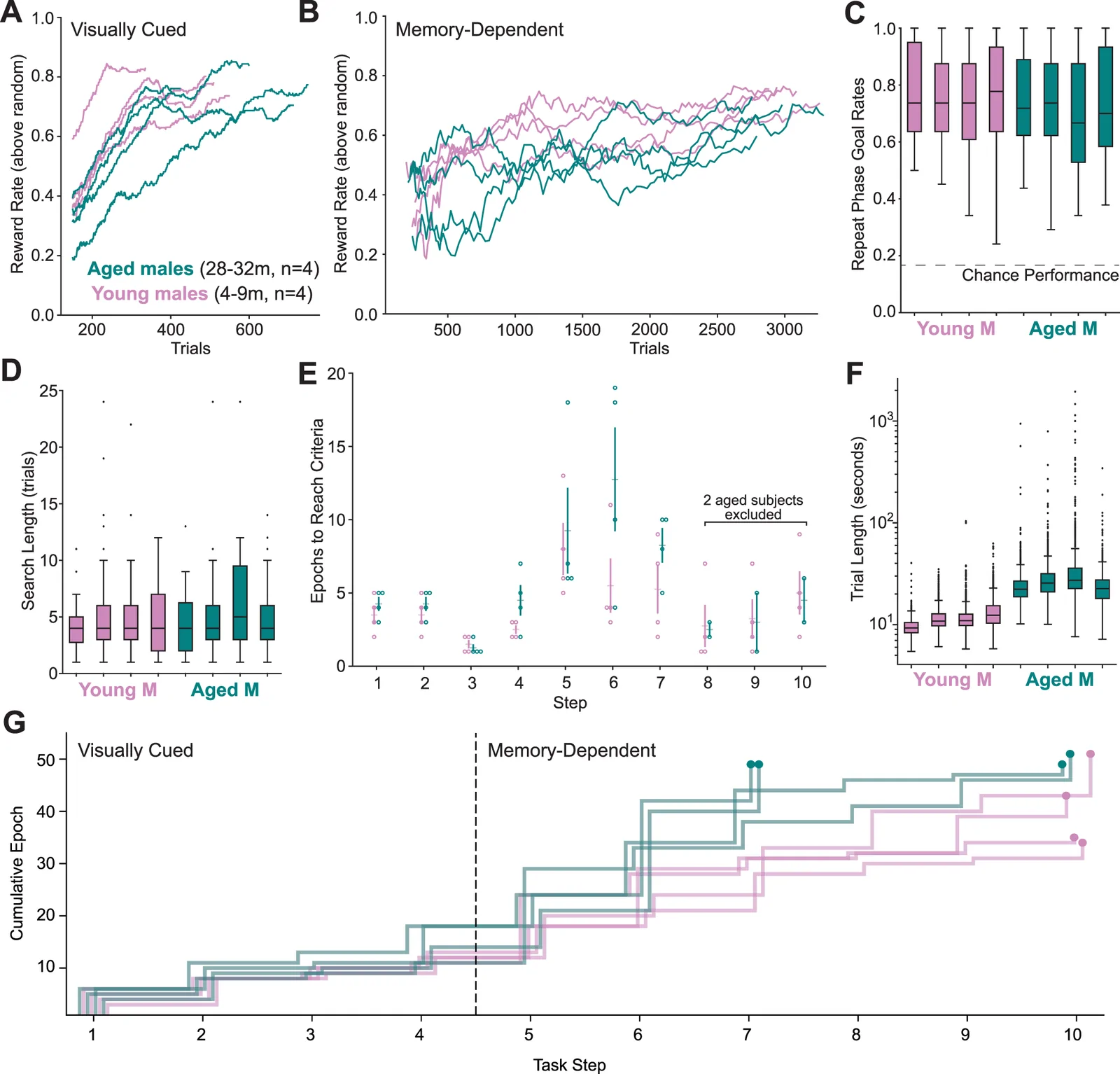
Male cohort results. ***A***, Learning curves by subject for the visually cued task steps. This plot shows the reward rate for each subject minus the reward rate a random agent would achieve, linearly normalized such that 0 is the reward rate of a random agent and 1 is the reward rate a subject would achieve with a 100% goal rate. Data was smoothed with a 150-trial moving average. *n*_trials_ = [*Y*1: 510, *Y*2: 550, *Y*3: 336, *Y*4: 490, *A*1: 600, *A*2: 424, *A*3: 713, *A*4: 750]. ***B***, Learning curves by subject for the memory-dependent task steps, linearly normalized such that 0 is the reward rate of a random agent (that only selects lit wells) and 1 is the reward rate a subject would achieve with a 100% goal rate. An overall reward rate was calculated for each goal block and then smoothed with a moving average of 10 goal blocks (this eliminates oscillation due to the search phase/repeat phase structure of the task). *n*_trials_ = [*Y*1: 3,303, *Y*2: 3,096, *Y*3: 3,498, *Y*4: 3,172, *A*1:3,206, *A*2: 3,537, *A*3: 3,044, *A*4: 2,990] (includes plateaued behavior). ***C***, Median reward rate after finding the goal (repeat phase) during “fully trained” memory-dependent epochs, after criterion was met for Step 10. Effect of age evaluated by linear mixed-effect model (Python statsmodels mixedlm function): *p* = 0.205, *n*_blocks_ = [*Y*1: 36, *Y*2: 70, *Y*3: 54, *Y*4: 68, *A*1: 32, *A*2: 35, *A*3: 31, *A*4: 39] (*p* value calculated using Powell's method, instead of the default BFGS optimization method for the mixed-effects linear regression). ***D***, Median length of search phase per goal block during “fully trained” memory-dependent epochs, after criterion was met for Step 10. Effect of age evaluated by linear mixed-effect model: *p* = 0.123, *n*_blocks_ = [*Y*1: 36, *Y*2: 70, *Y*3: 54, *Y*4: 68, *A*1: 32, *A*2: 35, *A*3: 31, *A*4: 39]. ***E***, Average number of training epochs required to meet progression criteria for each step; subjects excluded for steps they were unable to complete (panel ***G***). Ranksum results by step (Extended Data Fig. 6-1) do not demonstrate a significant effect of age on learning rates for this task in this small cohort of males. ***F***, Median length of time for each trial (time from initial home poke to the home poke initiating the next trial). Effect of age evaluated by linear mixed-effect model: *p* *<* 0*.*001*, n*_trials_ = [*Y*: 848, *Y*2: 1,711, *Y*3: 1,358, *Y*4: 1,672, *A*1: 781, *A*2: 961, *A*3: 909, *A*4: 1,010]. ***G***, Step plot showing the cumulative advancement of each male subject through the curriculum. Note that horizontal offsets are added so each individual subject line is visible. The circle at the end of each line indicates the furthest epoch completed by that subject prior to manual advancement.

The male cohort was trained using the curriculum shown in Figure 5*D*, with minor differences indicated by asterisks and detailed in the figure caption. For this cohort, subjects received a small reward at the home port and a large reward at outer arm ports. Subjects showed a rapid increase in reward rate during the visually cued epochs, all reaching performance levels well above chance, but also demonstrating substantial individual variability in learning rate and ultimate performance level (Fig. 6*A*). The memory-dependent version of the task was learned much more slowly, but ultimately all subjects reached performance rates well above chance (Fig. 6*B*). Two of our aged subjects failed to reach the set criterion for memory-dependent Step 8 after many epochs (10 epochs for A1 and eight epochs for A3) and were manually advanced to Step 9 for a single epoch, and then to Step 10. Despite this, we found no obvious differences in performance at the final task between aged subjects who completed the curriculum and aged subjects who were advanced manually. Additionally, we found no significant difference between age groups in repeat phase reward rate (Fig. 6*C*) or the length of the search phase; a measure indicative of search efficiency (Fig. 6*D*). The amount of training required to reach criterion for most steps was suggestive of slower learning in the aged subjects, particularly for the first few steps of the memory-dependent task variant (Fig. 6*E*,*G*), although this also did not reach statistical significance likely due to our small sample size (Extended Data Fig. 6-1). The most drastic difference between age groups was in trial duration (Fig. 6*F*), as aged subjects moved far more slowly than young subjects. Despite their slow speed, aged subjects generally completed the designated number of trials or goal blocks and only rarely timed out.

10.1523/ENEURO.0415-25.2026.f6-1 Figure 6-1 Male Ranksum Results. Download Figure 6-1, DOCX file.

**Figure 7.**
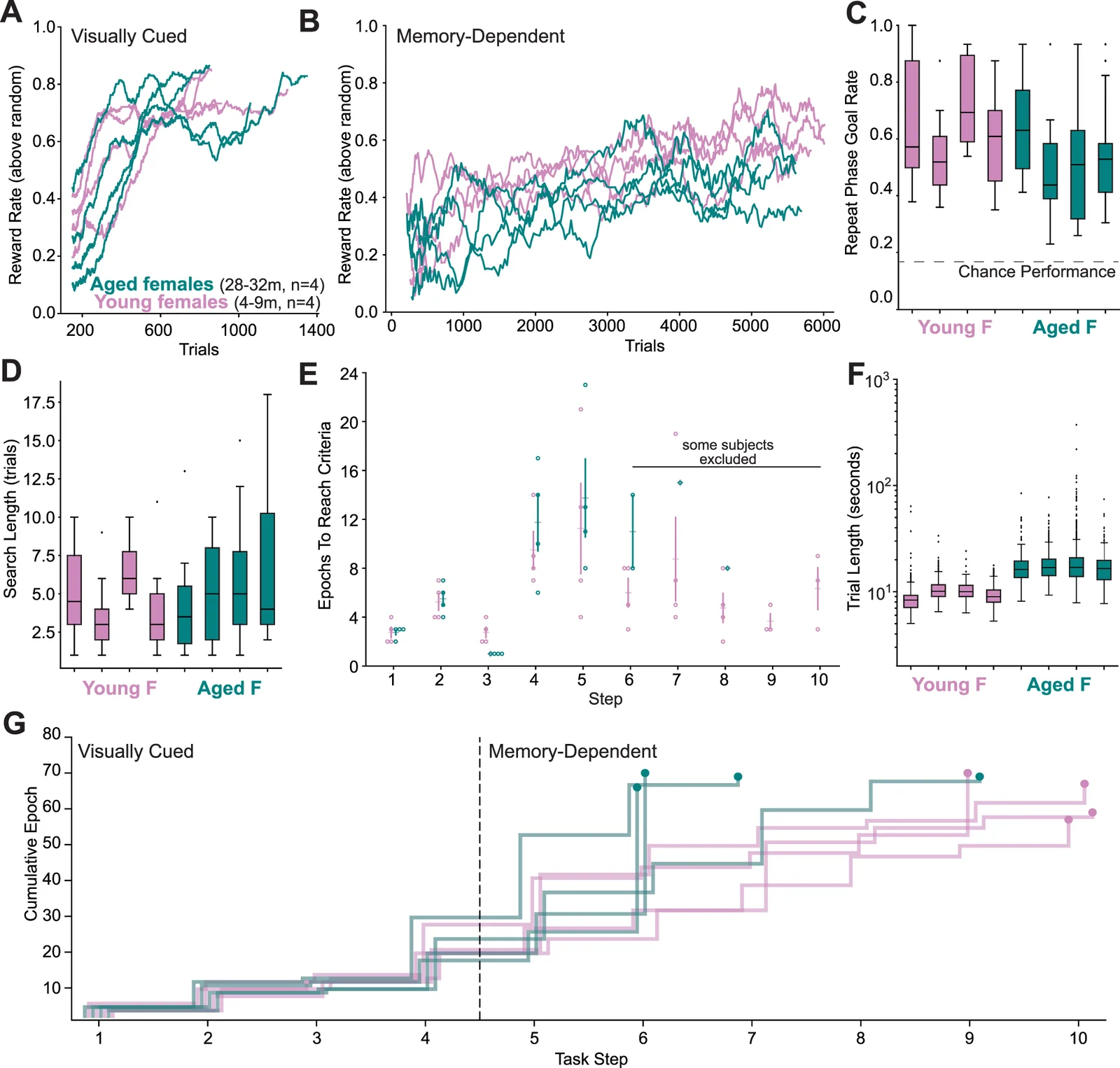
Female cohort results. ***A***, Learning curves by subject for the visually cued task steps. This plot shows the reward rate for each subject minus the reward rate a random agent would achieve, linearly normalized such that 0 is the reward rate of a random agent and 1 is the reward rate a subject would achieve with a 100% goal rate. Data was smoothed with a 150 trial moving average. *n*_trials_ = [*Y*1: 1,250, *Y*2: 910, *Y*3: 860, *Y*4: 840, *A1*: 1,060, *A*2: 750, *A*3: 850, *A*4: 1,350]. ***B***, Learning curves by subject for the memory-dependent task steps, linearly normalized such that 0 is the reward rate of a random agent (that only selects lit wells) and 1 is the reward rate a subject would achieve with a 100% goal rate. An overall reward rate was calculated for each goal block and then smoothed with a moving average of 10 goal blocks (this eliminates oscillation due to the search phase/repeat phase structure of the task). *n*_trials_ = [*Y*1: 5,675, *Y*2: 5,975, *Y*3: 6,135, *Y*4: 6,175, *A*1: 5,765, *A*2: 5,800, *A*3: 5,880, *A*4: 5,280] (includes plateaued behavior). ***C***, Median reward rate after finding the goal (repeat phase) during “fully trained” memory-dependent epochs, after criterion was met for Step 10. Effect of age evaluated by linear mixed-effect model (Python statsmodels mixedlm function): *p* = 0.046, *n*_blocks_ = [*Y*1: 12, *Y*2: 13, *Y*3: 6, *Y*4: 17, *A*1: 8, *A*2: 17, *A*3: 18, *A*4: 20] (*p* value calculated using Powell's method, instead of the default BFGS optimization method for the mixed-effects linear regression). ***D***, Median length of search phase per goal block during “fully trained” memory-dependent epochs, after criterion was met for Step 10. Effect of age evaluated by linear mixed-effect model: *p* = 0.097, *n*_blocks_ = [*Y*1: 12, *Y*2: 13, *Y*3: 6, *Y*4: 17, *A*1: 8, *A*2: 17, *A*3: 18, *A*4: 20]. ***E***, Average number of training epochs required to meet progression criteria for each step; subjects excluded for steps they were unable to complete (panel ***G***). Ranksum results for steps completed by at least two aged female subjects (Extended Data Fig. 7-1). Most steps did not demonstrate a significant effect of age on learning rates in this small female cohort, although aged subjects advanced through Step 3 slightly but significantly more quickly than young subjects. ***F***, Median length of time for each trial (time from initial home poke to the home poke initiating the next trial). Effect of age evaluated by linear mixed-effect model: *p* *<* 0*.*001, *n*_trials_ = [*Y*1: 420, *Y*2: 490, *Y*3: 210, *Y*4: 630, *A*1: 280, *A*2: 910, *A*3: 910, *A*4: 910]. ***G***, Step plot showing the cumulative advancement of each female subject through the curriculum. Note that horizontal offsets are added so each individual subject line is visible. The circle at the end of each line indicates the furthest epoch completed by that subject prior to manual advancement.

The female cohort was also trained using the curriculum shown in Figure 5*D*, with minor differences noted in the figure caption. Importantly, however, this cohort was provided only a small reward at both the home and outer arm ports, since the female rats were much smaller and more likely to satiate with the amount of milk reward provided to the male cohort. Perhaps as a result of this difference, the female cohort learned both the visually cued and memory-dependent task steps more slowly (Fig. 7*A*,*B*). Further, three aged subjects and one young subject were unable to meet the performance criteria required to advance through the full curriculum (Fig. 7*E*,*G*). These subjects were manually advanced to the final Step 10 task variant, where we saw that aged subjects had a significantly lower reward rate during the repeat phase (Fig. 7*C*) and trended toward longer search phases (indicative of inefficient searching; Fig. 7*D*). However, these results could reflect the altered relative reward amounts and/or the manual advancement rather than reflecting true differences in performance ability. Prior to becoming unable to advance, however, aged subjects showed a trend toward slower learning, especially in the memory-dependent steps, when compared to young subjects (Fig. 7*B*,*E*,*G*; Extended Data Fig. 7-1). They also moved far more slowly (Fig. 7*F*). Both of these results are consistent with the results of the male cohort.

10.1523/ENEURO.0415-25.2026.f7-1 Figure 7-1 Female Ranksum Results. Download Figure 7-1, DOCX file.

## Discussion

The results from the pilot cohorts demonstrate that the mazes perform as expected, achieving our major goal of training cohorts of rats with minimal experimenter interference on a complex curriculum. In contrast to prior training regimes for a similar task (Gillespie et al., 2021), our apparatus allowed us to train twice as many rats in about half the time. Further, our training process now allows us to capture different learning phases in a regimented and comparable manner. The gradual advancement through increasingly complex steps allowed more rats to continue participating, rather than being dropped from the cohort due to poor motivation or low performance. While aged subjects were certainly capable of learning and performing the final memory-dependent task variant, differences in movement speed and learning rate suggest that this is an appropriate task in which to study the impact of age on memory processes. Additionally, with experimenter-induced variability minimized, the individual variability remaining in both age groups further suggests that this task may be well suited to explore the neural basis of individual variability in cognitive performance.

Two aspects of the behavioral analysis were particularly illuminating. First, the dramatic difference in movement speed, and thus trial length, between the age groups for both sexes was striking. While unsurprising (Birren and Fisher, 1995; McQuail and Nicolle, 2015), this underscored the importance of having each epoch be limited by trial or goal block criteria, rather than by time (as is more common). This ensured that the aged and young subjects received similar amounts of experience with each epoch and will be essential for any studies of aged subjects. It was also encouraging that, when given sufficient time, aged subjects were motivated and able to engage with the task similarly to the young subjects. Second, the implementation of such a gradual curriculum was effective, as indicated by the lack of any stark performance drop-off caused by progression to a specific task step and the continued engagement of every subject throughout the curriculum, even for subjects who did not achieve all advancement criterion. Further, the inclusion of learning both a visually cued and memory-dependent task separates the learning of distinct task rules, allowing for future investigation into the neural correlates of each rule separately.

### Limitations

While very infrequent, hardware errors and experimenter errors did still occasionally occur. Hardware errors tended to be reward port failure due to beam break sensors failing or tubing leaks causing failure in reward delivery. These issues were difficult to catch by inspection of the apparatus but became readily apparent during behavior as subjects would rapidly learn to avoid ports that never delivered reward, and these biases were clearly visible on the live performance plots. Watching for these issues required attentiveness from the experimenter, but once caught were promptly fixed, and epochs were redone as necessary (dropping any data which was adversely affected). Experimenter error tended to be minor, and extra epochs were performed to replace impacted data.

A second limitation that our pilot cohorts revealed was the impact of setting advancement criteria at a level achievable by all subjects, while also enforcing sufficient learning of the current task variant. The two aged males who were unable to advance past Step 8 were still performing well above chance, and nearly at criterion—suggesting not that they failed to learn but that the criterion was set unreasonably high. Furthermore, after manual advancement, their final performance was not different than the other aged subjects. This strongly suggests that the issue was in fact one of parameter choice rather than a true performance difference. In the female cohort, we lowered the advancement criteria for memory-dependent steps but also introduced another change: we reduced the amount of reward provided at outer arm ports. We found that more female rats were unable to advance: three aged subjects and one young subject. We hypothesize that the reduction in reward amount may have substantially reduced motivation as the task variants became more difficult, obscuring any benefit of the lower advancement criterion. Another possibility is that lowering the outer reward amount caused subjects to devalue the outer arms and rely on the home well to provide reward, without learning to choose the outer arm optimally. In future behavioral studies, we will retain relatively larger reward amounts at the outer arm ports, and we may further refine the advancement criteria, potentially by incorporating metrics other than or in addition to simple overall reward rate. Future implementation of this task should give careful consideration to advancement criteria, especially considering the specific goals of the experiment. For instance, fixed advancement criteria may be essential for comparing learning between experimental cohorts, while more flexible criteria may be appropriate if the goal is to train all subjects to perform a final high-priority task variant. Since many factors (rat strain, age, experimental intervention, room setup, and more) may influence behavioral performance, more pilot training cohorts will be essential to inform performance expectations when developing new tasks or curricula.

### Future outlook

While the current apparatus substantially extends our ability to train and collect data on larger cohorts of animals, it is still far from truly high-throughput behavior as other groups have achieved (Poddar et al., 2013; Hao et al., 2021). One major difference is our continued use of discrete, spaced training sessions in specific maze environments separate from the home cage, which will always require more time and experimenter involvement than in-cage, unsupervised, continuous engagement tasks. Another key limitation to higher throughput is the requirement for overhead camera-based tracking throughout the entire maze. If position information were unnecessary, or could be collected via alternate means [via inertial sensors (Fayat et al. 2025), for instance], then it would be feasible to stack 3–4 mazes vertically and run 12–16 subjects simultaneously with the same spatial footprint. Some updates to drainage and cleaning strategies would be needed in this case, but all task control and logging software would seamlessly extend to the stacked use case. Such stacking of the mazes could allow comparable throughput to other highly successful behavioral training approaches (Poddar et al., 2013; Hao et al., 2021) and would be the first to our knowledge to achieve this level of throughput for a spatial navigation task.

Even at its current capacity, the maze apparatus opens up exciting possibilities for efficiently testing larger cohorts of male and female subjects, rodent models of disease, and performing longitudinal behavioral assessment of cohorts across the lifespan—all of which will be fascinating comparators to the expansion of our existing pilot study of age-related behavioral differences. Since the apparatus is also fully compatible with wireless neural recordings and overhead position tracking, future studies will include the assessment of neural activity patterns that underlie the learning and memory processes that we set out to study. The ability to test larger cohorts of animals with minimal preselection will allow us to capture and characterize performance across a much wider range of abilities, facilitating our understanding of the neural basis of individual performance differences. Another key challenge in studying individual behavioral differences is the need to identify biologically driven, rather than experimentally induced, variability. The automation and parallelization of the current maze apparatus suggests that the variability we see in pilot cohort performance may reflect, and allow the study of, true individual differences.

We hope that the low-cost and open-source nature of many aspects of our task apparatus will make it a useful resource for others striving to scale up and increase the automation of their behavioral testing approaches. In the described maze environment, the flexible nature of the task logic can allow for many different tasks to be implemented, such as sequence learning tasks, alternation tasks (Frank et al., 2000; Kastner et al., 2020), or even pure foraging tasks. The use of alternative maze environments equipped with our, or other, maze features (Porter et al., 2025) opens endless possibilities for structured, efficient behavioral testing. Regardless of behavioral paradigm, a shift toward parallelized training offers improved control of other influences on behavior, such as circadian rhythms, in addition to increased efficiency. Finally, the adoption of a structured curriculum with trial-based advancement criteria, rather than time-based advancement criteria, will be beneficial for accurately and rigorously capturing behavioral differences across groups of subjects no matter the species or task.

## Synthesis

Reviewing Editor: Mark Sheffield, University of Chicago Division of the Biological Sciences Decisions are customarily a result of the Reviewing Editor and the peer reviewers coming together and discussing their recommendations until a consensus is reached. When revisions are invited, a fact-based synthesis statement explaining their decision and outlining what is needed to prepare a revision will be listed below. The following reviewer(s) agreed to reveal their identity: Manu Madhav.

Overall, the manuscript presents a thoughtfully designed, automated, and accessible system for parallelized spatial navigation training in rats, with clear strengths in space efficiency, reduced experimenter intervention, and reliance on affordable, open-source components. The apparatus is clearly described, the design decisions are well motivated, and the system appears broadly replicable. The pilot training data demonstrate that both young and aged rats can successfully learn a multi-stage spatial task using this platform, supporting its practical feasibility.

At the same time, several central claims would benefit from clearer justification, quantitative support, or more careful framing. In particular, assertions regarding "higher throughput," flexibility, scalability, and low cost would be strengthened by explicit metrics (e.g., setup time, reconfiguration effort, and cost breakdowns) and/or direct comparison to conventional approaches, or alternatively by tempering these claims and acknowledging inherent constraints. The gains in throughput appear to derive primarily from parallelization and space optimization rather than continuous, unsupervised training, and this distinction should be made explicit. Relatedly, the system necessarily relies on spaced training sessions rather than continuous engagement, which differentiates it from cage-based high-throughput systems and introduces limitations that should be more clearly discussed.

Additional practical and conceptual limitations warrant explicit acknowledgement. The fully enclosed design, while effective for preventing escape and enabling reliable automation, constrains compatibility with tethered electrophysiology systems and may bias animals toward egocentric or intra-maze strategies. Because distal allocentric cues are likely occluded by the enclosure, spatial learning may rely predominantly on local or ceiling-mounted cues, potentially limiting the types of navigational strategies that can be trained. Clarifying the visibility of external cues, the role of ceiling cues, and the extent to which allocentric navigation can be supported, or is intentionally deprioritized, would strengthen the manuscript's conceptual positioning.

Several points of clarification and validation would further enhance rigor and reproducibility. Including photographs of the physical apparatus, in addition to rendered figures, would help readers better assess design features such as the mesh ceiling and enclosure geometry. Given the stated goal of compatibility with wireless electrophysiology, providing example neural recordings, particularly traces aligned to pump activation, would substantiate claims of minimal electrical or mechanical interference. Potential cross-maze interference, such as acoustic cues from neighboring animals, should also be addressed experimentally or discussed as a limitation with possible mitigation strategies.

Finally, aspects of the behavioral implementation and cohort design could be more fully justified. Fixed performance thresholds may unnecessarily constrain progression for some animals, suggesting that more flexible advancement criteria could improve robustness. The exclusive use of male rats requires justification, particularly in light of statements regarding future applications to female cohorts, which currently lack empirical support or clear rationale. Providing additional methodological details, such as the equations governing arm-balancing algorithms and clarification of camera access and monitoring at the central station, would further improve transparency.

## Author Response

Dear Dr. Sheffield, We are excited to submit a revised version of our manuscript to eNeuro. We are grateful for the general appreciation for our task design and scaling approaches, and our manuscript has certainly been improved by addressing the thoughtful suggestions from the reviewers. We're also pleased to be able to incorporate recently collected behavioral data from a female cohort as well as preliminary results from a cohort of subjects with electrophysiological recording implants performing the behavioral task. Below, please find a detailed point-by-point response to the reviewer comments. As instructed, we are including a version of the manuscript with the relevant edited/new content in bold as well as a clean copy including the title page. Please note that the figures included separately as .eps files have improved resolution (especially noticeable in the photographs) compared to those included in the manuscript pdf.

Thank you for your patience and consideration of the revised manuscript; we look forward to hearing from you.

-------------------------------------------------------------------------------------------------------------------------------- Synthesis of Reviews:

Overall, the manuscript presents a thoughtfully designed, automated, and accessible system for parallelized spatial navigation training in rats, with clear strengths in space efficiency, reduced experimenter intervention, and reliance on affordable, open-source components. The apparatus is clearly described, the design decisions are well motivated, and the system appears broadly replicable. The pilot training data demonstrate that both young and aged rats can successfully learn a multi-stage spatial task using this platform, supporting its practical feasibility.

At the same time, several central claims would benefit from clearer justification, quantitative support, or more careful framing. In particular, assertions regarding "higher throughput," flexibility, scalability, and low cost would be strengthened by explicit metrics (e.g., setup time, reconfiguration effort, and cost breakdowns) and/or direct comparison to conventional approaches, or alternatively by tempering these claims and acknowledging inherent constraints. The gains in throughput appear to derive primarily from parallelization and space optimization rather than continuous, unsupervised training, and this distinction should be made explicit. Relatedly, the system necessarily relies on spaced training sessions rather than continuous engagement, which differentiates it from cage-based high-throughput systems and introduces limitations that should be more clearly discussed.

We appreciate this feedback and have made some edits that should both strengthen some of our claims by adding some more explicit metrics and better acknowledge the constraints of the type of behavioral solution we are offering. The reviewer is absolutely correct that our increased throughput is achieved by parallelization and not by continuous engagement as used in other high-throughput systems. We have made this more explicit (Introduction, paragraph 4) and reconfigured some citations (Introduction, paragraphs 3/4) to make more clear which aspects of other behavioral tasks we are emulating. In addition, we further described the limitations of this approach compared to continuous engagement tasks (Discussion, Future Outlook, paragraph 1). To provide more explicit metrics reflecting the low cost and scalability of our apparatus, we now include a dedicated section discussing cost, assembly time, and comparisons to other options (Materials and Methods, section 2.6).

Additional practical and conceptual limitations warrant explicit acknowledgement. The fully enclosed design, while effective for preventing escape and enabling reliable automation, constrains compatibility with tethered electrophysiology systems and may bias animals toward egocentric or intra-maze strategies. Because distal allocentric cues are likely occluded by the enclosure, spatial learning may rely predominantly on local or ceiling-mounted cues, potentially limiting the types of navigational strategies that can be trained. Clarifying the visibility of external cues, the role of ceiling cues, and the extent to which allocentric navigation can be supported, or is intentionally deprioritized, would strengthen the manuscript's conceptual positioning.

The reviewer makes an excellent point here. Indeed, the enclosed space of the maze could encourage subjects to rely on local intra-maze cues and engage egocentric rather than allocentric strategies. To better convey the availability of cues, we now better describe the cues available to rat subjects (Materials and Methods, paragraph 1), include a picture of the mesh ceiling so that readers can appreciate how minimally it occludes ceiling visibility (Figure 2F), and include a rat's-eye view photograph which shows prominent local and distal ceiling cues (Extended Data Figure 1C).

Several points of clarification and validation would further enhance rigor and reproducibility. Including photographs of the physical apparatus, in addition to rendered figures, would help readers better assess design features such as the mesh ceiling and enclosure geometry. Given the stated goal of compatibility with wireless electrophysiology, providing example neural recordings, particularly traces aligned to pump activation, would substantiate claims of minimal electrical or mechanical interference. Potential cross-maze interference, such as acoustic cues from neighboring animals, should also be addressed experimentally or discussed as a limitation with possible mitigation strategies.

We completely agree that photos and videos can provide a much clearer sense of the maze apparatus. We now include an overall photo of the 4-maze configuration (Extended Data Figure 1A), a close-up photograph of one of the mazes (Fig 2A), a closeup photograph of the maze lid (Fig 2F), and a photo of the command center screens (Extended Data Figure 1B), which shows all the information available to the experimenter to monitor task performance during behavior epochs. In addition, we now include a movie showing the 4 mazes operating simultaneously (Supplementary Movie 1). Finally, we also include two example overhead videos taken from the mazes to show what visual information would be available to position tracking software (Supplementary Video 2, 3).

To demonstrate the compatibility of the maze apparatus with neurophysiology, we now include a figure (Extended Data Figure 4) showing electrophysiological recordings from the hippocampus during behavior in the maze. To give the clearest sense of any induced artifact (or lack thereof), we show two representative examples of unreferenced neural traces aligned to the times of pump activation, one at an outer arm port and the other at the home port. These traces demonstrate that the pumps do not induce electrical artifacts.

The reviewer also makes a good observation that cross-maze interference is possible with the current apparatus. We now include a more explicit note that indeed each maze is not acoustically isolated, but that this could likely be achieved if necessary with sound isolating foam walls installed between mazes and/or around each PER (Materials and Methods Section 2.4, paragraph 3). We also include a video that includes the sound of the pumps during behavior (Supplementary Movie 1) so that readers can get a sense of how quiet the pump activity is and evaluate for themselves the reaction of the subjects running behavior.

Finally, aspects of the behavioral implementation and cohort design could be more fully justified. Fixed performance thresholds may unnecessarily constrain progression for some animals, suggesting that more flexible advancement criteria could improve robustness. The exclusive use of male rats requires justification, particularly in light of statements regarding future applications to female cohorts, which currently lack empirical support or clear rationale. Providing additional methodological details, such as the equations governing arm-balancing algorithms and clarification of camera access and monitoring at the central station, would further improve transparency.

The reviewer is correct that the exclusive use of male rats was a major limitation to our prior manuscript version. We are pleased to now include cohorts of both male and female rats at aged and young timepoints, as it was important to be able to show that both sexes would behave in the maze apparatus. The female data is now described in the Results section (paragraphs 1 and 3) and included as Extended Data Figure 6. We have also added an additional panel for both the male (Figure 6G) and the female figures (Extended Data Figure 6G) showing the cumulative progression through each task step, which gives a better sense of age differences over the full curriculum.

We also appreciate the reviewer's observation that the fixed performance thresholds are arbitrary and could constrain progression for some animals. Indeed, the thresholds did prevent progression for some individuals from both the male and female cohorts as discussed in Results, paragraphs 2 and 3, now more clearly illustrated for the male cohort in Figure 6G and for the female cohort in Extended Data Figure 6G, and more extensively discussed in the Discussion (Limitations, paragraph 2) . On one hand, having a fixed criteria is beneficial for our ability to compare between individuals or cohorts ("how many epochs are required to reach x performance level?"), but of course they also must be set carefully to not arbitrarily exclude subjects from advancing and prevent data collection during high-priority task variants. The advancement criteria is an area of ongoing optimization, which we now more clearly acknowledge in the Discussion (Limitations, paragraph 2). Since many possible factors could introduce differences between our results and replicates in other labs, we now include an explicit suggestion that if readers use our 6-arm task curriculum, they test the advancement criteria and ensure that it is optimized for the specific goals of their experiment (ie comparing learning rate between cohorts vs training all subjects to reach a final high-priority task variant).

To improve the transparency of our methods, we now include the arm-balancing algorithms in Appendix A, as suggested. In addition, we hope that methodological details will be made more clear with the inclusion of photographs (Extended Data Figure 1, Figure 2). In addition to an overall video showing all mazes performing simultaneously, we now also include two example videos from the overhead cameras, which will clarify camera access and demonstrate the video quality and subject visibility that would be available for any behavioral tracking software.
