# Supplementary material for: A Parallelized, Automated System for Higher-Throughput Spatial Navigation Testing of Rats
Source: eNeuro. 2026 Aug 21;13(8):ENEURO.0415-25.2026. doi: 10.1523/ENEURO.0415-25.2026 (PMC13505593; doi:10.1523/ENEURO.0415-25.2026)
Supplement: Supplementary file 1 [file eneuro-13-ENEURO.0415-25.2026-s004.docx]

**Figure 6-1. Male Ranksum Results**

| step | 1 | 2 | 3 | 4 | 5 | 6 | 7 | 8 | 9 | 10 |
| --- | --- | --- | --- | --- | --- | --- | --- | --- | --- | --- |
| n*young* | 4 | 4 | 4 | 4 | 4 | 4 | 4 | 4 | 4 | 4 |
| n*aged* | 4 | 4 | 4 | 4 | 4 | 4 | 4 | 2 | 2 | 2 |
| ranksum statistic | -0.866 | -0.866 | 0.577 | -1.44 | -0.289 | -1.44 | -1.44 | -0.694 | 0.231 | 0.00 |
| p value | 0.39 | 0.38 | 0.56 | 0.15 | 0.77 | 0.15 | 0.15 | 0.49 | 0.82 | 1.0 |
