# Supplementary material for: A Parallelized, Automated System for Higher-Throughput Spatial Navigation Testing of Rats
Source: eNeuro. 2026 Aug 21;13(8):ENEURO.0415-25.2026. doi: 10.1523/ENEURO.0415-25.2026 (PMC13505593; doi:10.1523/ENEURO.0415-25.2026)
Supplement: Supplementary file 2 [file eneuro-13-ENEURO.0415-25.2026-s005.docx]

**Figure 7-1. Female Ranksum Results**

| step | 1 | 2 | 3 | 4 | 5 | 6 | 7 | 8 | 9 | 10 |
| --- | --- | --- | --- | --- | --- | --- | --- | --- | --- | --- |
| n*young* | 4 | 4 | 4 | 4 | 4 | 4 | 4 | 4 | 4 | 4 |
| n*aged* | 4 | 4 | 4 | 4 | 4 | 2 | 1 | 1 | 0 | 0 |
| ranksum statistic | -0.144 | -0.289 | 2.309 | -0.722 | -0.722 | -1.389 | - | - | - | - |
| p value | 0.885 | 0.773 | 0.021 | 0.470 | 0.470 | 0.165 | - | - | - | - |
